# Sex-specific associations of kynurenic acid with neopterin in Alzheimer’s disease

**DOI:** 10.1186/s13195-024-01531-7

**Published:** 2024-07-27

**Authors:** Anne-Brita Knapskog, Trine Holt Edwin, Per Magne Ueland, Arve Ulvik, Evandro Fei Fang, Rannveig Sakshaug Eldholm, Nathalie Bodd Halaas, Lasse M. Giil, Ingvild Saltvedt, Leiv Otto Watne, Mari Aksnes

**Affiliations:** 1 Department of Geriatric Medicine, Oslo University Hospital, 0450 Oslo, Norway; 2 Bevital AS, 5021 Bergen, Norway; 3 Department of Clinical Molecular Biology, University of Oslo and Akershus University Hospital, 1478 Lørenskog, Norway; 4 The Norwegian Centre On Healthy Ageing (NO-Age), Oslo, Norway; 5 Department of Neuromedicine and Movement Science, Norwegian University of Science and Technology, 7491 Trondheim, Norway; 6 Department of Geriatric Medicine, St. Olavs Hospital, Trondheim University Hospital, 7006 Trondheim, Norway; 7 Oslo Delirium Research Group, Oslo University Hospital, 0450 Oslo, Norway; 8 Department of Geriatric Medicine, University of Oslo, 0315 Oslo, Norway; 9 Neuro-SysMed, Department of Internal Medicine, Haraldsplass Deaconess Hospital, 5892 Bergen, Norway; 10 Department of Clinical Science, University of Bergen, 5021 Bergen, Norway; 11 Institute of Clinical Medicine, Campus Ahus, University of Oslo, 1478 Lørenskog, Norway; 12 Department of Geriatric Medicine, Akershus University Hospital, 1478 Lørenskog, Norway

**Keywords:** Alzheimer disease, Biomarkers, Cerebrospinal fluid, Kynurenic acid, Kynurenine pathway, Longitudinal case control study, Quinolinic acid, Sex differences, Tryptophan, Mitophagy

## Abstract

**Background:**

Sex differences in neuroinflammation could contribute to women’s increased risk of Alzheimer’s disease (AD), providing rationale for exploring sex-specific AD biomarkers. In AD, dysregulation of the kynurenine pathway (KP) contributes to neuroinflammation and there is some evidence of sex differences in KP metabolism. However, the sex-specific associations between KP metabolism and biomarkers of AD and neuroinflammation need to be explored further.

**Methods:**

Here we investigate sex differences in cerebrospinal fluid concentrations of seven KP metabolites and sex-specific associations with established AD biomarkers and neopterin, an indicator of neuroinflammation. This study included 311 patients with symptomatic AD and 105 age-matched cognitively unimpaired (CU) controls, followed for up to 5 years.

**Results:**

We found sex differences in KP metabolites in the AD group, with higher levels of most metabolites in men, while there were no sex differences in the CU group. In line with this, more KP metabolites were significantly altered in AD men compared to CU men, and there was a trend in the same direction in AD women. Furthermore, we found sex-specific associations between kynurenic acid and the kynurenic acid/quinolinic acid ratio with neopterin, but no sex differences in the associations between KP metabolites and clinical progression.

**Discussion:**

In our cohort, sex differences in KP metabolites were restricted to AD patients. Our results suggest that dysregulation of the KP due to increased inflammation could contribute to higher AD risk in women.

**Supplementary Information:**

The online version contains supplementary material available at 10.1186/s13195-024-01531-7.

## Background

Neuroinflammation is a central feature of Alzheimer’s disease (AD) pathogenesis [1], and sex differences in immune function is a possible driver of women’s increased risk of AD [2]. Dysregulation of the kynurenine pathway (KP) is present with neuroinflammation and occurs in AD and other neurodegenerative diseases [3–6]. Importantly, a disrupted KP contributes to a wide range of pathophysiological processes, including excitotoxicity, oxidative stress, compromised NAD^+^ synthesis, tau phosphorylation and amyloid aggregation [7]. In general, KP activity appears to be lower in women than in men and some studies have reported sex-specific alterations of KP metabolites in AD, but associations with neuroinflammation and AD pathophysiology must be explored further [8, 9].

The KP produces a series of intermediate metabolites, collectively referred to as kynurenines, which could be neuroprotective, *e.g.* kynurenic acid (KA) and picolinic acid (Pic), or neurotoxic, *e.g.* 3-hydroxykynurenine (3-HK), 3-hydroxyanthranilic acid (3-HAA), and quinolinic acid (QA) [7, 10, 11]. QA and KA are key neuroactive metabolites with respectively agonistic and antagonistic effects on the *N*-methyl-*D*-aspartate (NMDA) receptor, a pivotal receptor in neuronal activity and AD progression [12, 13]. High levels of QA induce excitotoxic damage by mimicking the actions of glutamate on the NMDA-receptor [14]. KA may reduce excitotoxicity through its antagonistic binding of the NMDA-receptor, but also affects the immune response by antagonistic effects on the α7 nicotinic acetylcholine receptor of the cholinergic system [15].

Studies of kynurenines in AD have provided mixed results. Most studies have assessed KP metabolites in blood, while levels in the cerebrospinal fluid (CSF) are less explored; correlations between these mediums vary from weak (KA) to strong (QA) [16]. While KP metabolites appear to be involved in AD pathophysiology, their relation to core AD biomarkers such as CSF levels of amyloid-β_42_ (Aβ_42_) and phosphorylated tau_181_ (p-tau_181_) are unclear; some have reported associations with Aβ_42_ and p-tau_181_ [17–19], while others found no associations with Aβ_42_ [18–21].We recently showed that the neuroprotective metabolites KA and Pic were increased in the CSF in AD compared to age-matched controls and that high levels of KA were associated with slower clinical progression [19], suggesting that increased KA production could be an adaptive response to neuronal damage. In healthy controls, increased plasma 3-HAA/AA ratios at baseline have recently been shown to predict risk of progression to mild cognitive impairment (MCI) or dementia [22].

Accumulating evidence suggests that dysregulation of the KP is involved in AD pathophysiology together with neuroinflammation. Concurrently, emerging data suggest that the well-established sex differences in systemic immune responses also extend to the central nervous system (CNS) [23, 24]. Sex-specific studies of microglia, the immune cells of the CNS, suggest that microglial activation is more closely tied to Aβ pathology in females compared to males [25, 26]. Notably, the kynurenine 3-HK and its downstream metabolites, including QA, are formed in the microglia [27]. Therefore, KP metabolites might be useful biomarkers of sex differences in AD pathophysiology and progression, but few studies have assessed sexual dimorphism in KP metabolism in relation to AD. While higher concentrations of KA and lower concentrations of tryptophan have been observed in male AD-model mice [28], higher KA and QA in CSF of AD patients compared to controls and higher serum kynurenine and AA in amyloid positive cognitively unimpaired (CU) individuals have been observed exclusively in women [20, 29]. One study of AD patients using the pooled CSF concentrations of tryptophan and several KP metabolites (kynurenine, 3-HK, KA, XA and QA) found no sex differences [6], whereas studies of individual KP metabolites have revealed sex differences in blood and CSF [9, 18]. In sum, there is a need to explore the effect of sex on KP metabolites in large well-characterised cohorts.

Plausibly, sex differences in KP metabolites are linked to differences in microglia and CNS immune responses. However, it is necessary to explore sex-specific associations between KP metabolites and indicators of immune responses. One candidate marker is neopterin, an established marker of immune activation and neuroinflammation [30, 31]. Both neopterin production and KP metabolism are stimulated by the cytokine interferon-γ (IFN-γ) [32, 33], and sex-specific associations between these markers may thus indicate upstream sex differences in inflammatory IFN-γ activity.

The current study aims to establish sex differences in CSF concentrations of KP metabolites and their associations with clinical progression by comparing men and women in a large well-characterised cohort consisting of patients with symptomatic AD and CU controls. Further, we aim to explore putative associations with AD pathophysiology and inflammation by assessing sex-specific interactions between KP metabolites, AD biomarkers and neopterin.

## Methods

### Study design

In this longitudinal study, we included patients from two Norwegian memory clinics at Oslo University Hospital in Oslo (n = 178), and at St. Olavs Hospital, Trondheim University Hospital in Trondheim (n = 133). Additionally, 105 CU controls were included.

#### Patients with AD

We recruited 311 patients from the Norwegian Registry of Persons Assessed for Cognitive Symptoms (the NorCog registry) from 2010 to 2018. A comprehensive assessment based on a standardised research protocol was conducted [34]. This included an interview with patients and their caregiver(s); cognitive testing; a physical examination; CSF and blood sampling; and imaging with computed tomography, magnetic resonance imaging, or fluorodeoxyglucose-positron emission tomography. At the time of inclusion, 28 patients (9%, 20 women) used symptomatic anti-dementia drugs; patients not on medication were given these drugs during follow up when applicable.

All patients fulfilled the criteria from the National Institute on Aging and the Alzheimer’s Association [35, 36] of probable or possible AD or AD combined with cerebrovascular disease; patients with other mixed pathologies were not included. Fifty-nine patients were at the stage of MCI, and 252 were at the dementia stage. The majority had mild dementia. In patients with a minimum of one clinical follow-up after baseline (n = 238), progression was evaluated with the Clinical Dementia Rating (CDR) scale. The degree of cognitive and functional impairment was estimated post hoc by certified CDR raters (Knight Alzheimer Disease Research Center certification) based on all clinical information, including activities of daily living and results of cognitive tests, from the patients’ records at baseline and at every follow-up consultation. The categories of memory, orientation, judgment and problem-solving, community affairs, home and hobbies, and personal care were scored on a 4-point scale ranging from 0 to 3, with higher values indicating more severe impairment. The scores for all categories were summarised to yield the CDR sum of boxes (CDR-SB), ranging from 0 to 18 [37]. Patients were followed for up to 5 years.

#### CU controls

The 105 CU controls were recruited in connection with preoperative evaluation for elective surgery involving spinal anaesthesia for gynaecological, orthopaedic, or urological conditions. Their CSF was collected at the onset of spinal anaesthesia, before anaesthetic agents were administered. Before surgery, the CU controls went through the same cognitive test battery as the AD patients. The CU controls were followed by repeated cognitive testing annually for up to 11 years. For more information about this cohort see Idland et al., 2020 [38].

### AD core biomarkers

#### Patients with AD

For the AD patients, the CSF AD core biomarkers CSF Aβ_42_ and p-tau_181_ were analysed at Akershus University Hospital in Nordbyhagen, Norway, using enzyme-linked immunosorbent assays (ELISA; Innotest ELISA hTau Ag, phoshoTau [181P] and β-amyloid 1–42 Fujirebio Europe, Ghent, Belgium). The laboratory’s own cut-off points were used. The results were considered abnormal when Aβ_42_ < 700 pg/ml and p-tau_181_ > 80 pg/ml for all patients [39].

#### CU controls

For the CU controls, the CSF AD core biomarkers were analysed at Sahlgrenska University Hospital in Mölndal, Sweden, using Innotest ELISAs with laboratory-specific pathological cut-off values: Aβ_42_ < 530 pg/ml and p-tau_181_ > 60 pg/ml [40].

### KP metabolites and neopterin

After collection in sterile cryotubes, the CSF samples were centrifuged for 10 min at 2,000 g, allocated into 0.5 ml cryotubes, and directly frozen at − 20 °C. Further, the samples were transported to the − 80 °C biobank for long-term cryopreservation within a week. Following one freeze-and-thaw cycle, the kynurenine metabolites were measured with liquid chromatography–tandem mass spectrometry at Bevital A/S in Bergen, Norway [41]. Tryptophan and eight KP metabolites (kynurenine, KA, AA, 3-HK, xanthurenic acid (XA), 3-HAA, Pic, and QA) were analysed together with neopterin. For the assay, the lower limits of detection ranged from 0.4 nmol/L (KA) to 8 nmol/L (Pic). The within day coefficients of variance ranged from 3.1% (Tryptophan) to 7.4% (AA) and the between day coefficients of variance ranged from 4.5% (3-HK) to 9.5% (AA).

### Interferon-γ induced protein 10

For all CU controls and a subset of the AD patients (n = 187) CSF levels of interferon-γ induced protein 10 (IP-10) were analysed as an additional indicator of IFN-γ activity, see Supplementary Methods.

### Statistical analyses

Statistical analyses were performed in IBM SPSS 29.0 and STATA 16.1. Data visualisations were created in STATA and R4.1.1 using RStudio and the split-violin script from Nordmann et al., [42].

The KP metabolites were log-transformed prior to parametric analyses to accommodate the distributional assumption of these analyses and as they followed log-normal distributions. All metabolites, continuous independent variables and continuous covariates were z-scored prior to regression and mixed model analyses. For all analyses two-sided *P* values less than 0.01 were considered statistically significant; corrections for multiple comparisons were not applied.

The metabolites were detectable in all CSF samples except for XA and 3-HAA, which were below the limit of detection (0.5 and 2 nM) in 100% and 51% of the samples (*i.e*. 84% among CU controls and 40% among AD patients), respectively. Hence, XA and 3-HAA were excluded from further statistical analyses. As the core biomarkers of AD in CSF were analysed at two laboratories, the results were dichotomised as normal or pathological in all analyses of samples from both patients and controls, *i.e.* analyses of the whole cohort. In analyses including only the AD patients or only the CU controls, CSF biomarkers were included as continuous variables.

#### Cross-sectional analyses

We used the Mann–Whitney U test for comparison of continuous variables, Pearson’s χ^2^ test for comparison of categorical variables, and Spearman’s ρ for correlation analyses.

To explore sex-specific associations between AD biomarkers, neopterin and KP metabolites, we performed regression analyses with tryptophan, KP metabolites and the KA/QA ratio as dependent variables. For the whole cohort (both AD patients and CU controls) we ran the following model: *metabolite* = *age* + *sex* + *diagnosis* + *APOE ε4-carrier (yes/no)* + *Aβ*_*42*_*-pathological (yes/no)* + *p-tau*_*181*_*-pathological (yes/no)* + *biomarker × sex.* For the separate analyses of AD patients and CU controls, we ran the following model: *metabolite* = *age* + *sex* + *CSF Aβ*_*42*_* (continuous)* + *CSF p-tau*_*181*_* (continuous)* + *biomarker × sex.* The biomarker × sex interactions Aβ_42_ × sex, p-tau^181^ × sex and neopterin × sex were included in separate models. For the CU controls and subset of AD patients with IP-10 measurements, models examining sex × IP-10 interactions were also performed. To explore the effects of body composition, models were rerun including body mass index (BMI) as a covariate for a subset of the AD patients with known BMI at baseline (n = 258); BMI at baseline was not available for the CU controls. Significant interactions were visualised in STATA using marginsplot.

#### Longitudinal analyses

To estimate the association of sex and baseline KP metabolite levels or the KA/QA ratio with clinical progression, we applied eight linear mixed-effects models with CDR-SB scores as the outcome variable and an unstructured covariance structure. The fixed effects included each metabolite or ratio, sex and time and were entered in the model as a 3-way interaction together with age: *CDR-SB* = *metabolite* + *sex* + *time* + *metabolite × sex* + *metabolite × time* + *sex × time* + *metabolite × sex × time* + *age.* Random effects included time and intercept. The time variable was calculated as the time between the baseline assessment and the CDR assessment (mean = 2.0 years, standard deviation = 1 year); to limit survival bias time was restricted to four years.

#### Sensitivity analyses

All analyses were rerun excluding the patients on anti-dementia medication (n = 28).

## Results

### Sample characteristics by sex

**Fig. 1:**
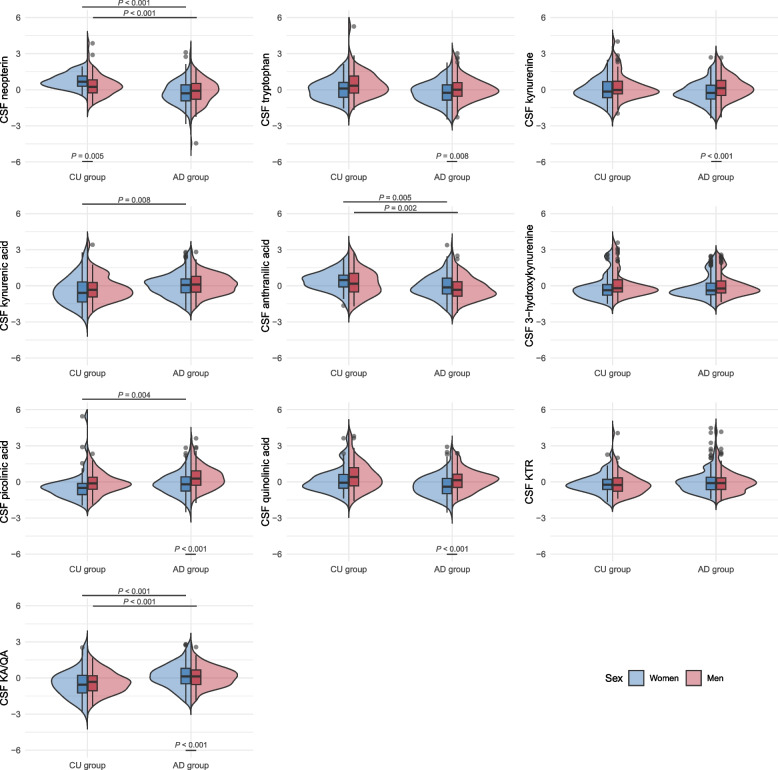
Distribution of z-scored CSF neopterin, tryptophan, kynurenines and KTR- and KA/QA ratios for men and women in the CU and AD groups. The split violin plots show the distribution, inserted box plots show the median (middle line), 1st quartile (lower box limit) and 3rd quartile (upper box limit). Abbreviations: AD: Alzheimer’s disease; KA/QA: kynurenic acid/quinolinic acid ratio; KTR: kynurenine/tryptophan ratio; CSF: cerebrospinal fluid; CU: cognitively unimpaired

Men and women did not differ in terms of age, frequency of *APOE* ε4 carriers, levels of AD biomarkers or frequency of pathological CSF AD core biomarkers in cross-sectional analyses, see Table 1. As expected, there was a significantly higher frequency of *APOE* ε4 carriers and pathological levels of CSF AD core biomarkers in the AD women and AD men compared to the CU women and CU men. CU women had significantly higher levels of neopterin (median = 30.6 nM) compared to CU men (median = 27.1 nM, *P* = 0.005) and AD women (median = 23.2 nM, *P* < 0.001). CU men had significantly higher levels of neopterin than AD men (median = 24.7, *P* < 0.001). There were no sex differences in IP-10 levels, but AD patients had significantly higher levels than CU controls, see Table 1. For a detailed comparison of the clinical characteristics of the AD patients and CU controls not stratified by sex, see Knapskog et al., 2023 [19]. Distributions of (standardised) CSF neopterin, tryptophan, kynurenines, as well as the KTR- and KA/QA ratios across men and women in the CU and AD group are presented in Fig. 1.

**Table 1:** Characteristics of the whole cohort by sex and diagnosis

	**AD women**	**AD men**		**CU women**	**CU men**		**AD-CU women**	**AD-CU men**
**Patient characteristics**			***P***			***P***	***P***	***P***
N	180 (57.9)	131 (42.1)		47 (44.8)	58 (55.2)			
Age, years	70.0 (66.0; 74.0)	72.0 (68.0; 75.0)	0.05	71.0 (69.0; 75.0)	70.0 (66.8; 76.0)	0.40	0.046	0.82
BMI^a^	23.3 (20.2; 27.2)	24.8 (22.9; 27.5)	**0.008**	NA	NA			
*APOE ε4* positive^a^ N (%)	125 (78.1)	83 (70.3)	0.14	20 (44.4)	17 (30.9)	0.16	** < 0.001**	** < 0.001**
**CSF AD core biomarkers**								
Amyloid-β_42_ (pg/ml)	549.0 (470.8; 650.8)	570 (450.0; 670.0)	0.82	702.0 (513.0; 830.0)	765.5 (580.8; 878.8)	0.34	†	†
Phosphorylated tau_181_ (pg/ml)	92.0 (64.0; 116.8)	86.0 (60.0; 106.0)	0.18	58.0 (45.0; 70.0)	54.0 (41.5; 66.8)	0.48	†	†
**Pathological CSF AD core biomarkers**								
Amyloid-β_42,_ N(%)	149 (82.8)	105 (80.2)	0.56	13 (27.7)	13 (22.4)	0.54	** < 0.001**	** < 0.001**
Phosphorylated tau_181,_ N(%)	108 (60.0)	74 (56.5)	0.54	20 (42.6)	21 (36.2)	0.51	0.03	**0.01**
**CSF inflammatory markers**								
Neopterin (nM)	23.2 (19.3; 28.9)	24.7 (20.0; 29.5)	0.23	30.6 (27.2; 35.1)	27.1 (23.1; 32.2)	**0.005**	** < 0.001**	**0.004**
IP-10 (ng/mL)^a^	1.9 (1.4; 2.4)	1.8 (1.5; 2.5)	0.37	1.3 (1.1; 1.9)	1.2 (0.9; 1.6)	0.13	** < 0.001**	** < 0.001**
**CSF kynurenine metabolites**								
Tryptophan (µM)	2.5 (2.2;2.9)	2.7 (2.4; 3.0)	**0.008**	2.7 (2.3; 3.1)	2.9 (2.5; 3.4)	0.045	0.06	0.02
Kynurenine (nM)	49.9 (41.8; 61.9)	56.9 (46.5; 70.9)	** < 0.001**	51.9 (43.7; 70.3)	54.2 (48.3; 69.6)	0.34	0.15	0.93
Kynurenic acid (nM)	3.5 (2.5; 4.7)	3.7 (2.6; 5.3)	0.33	2.5 (1.7; 4.1)	2.9 (2.1; 4.1)	0.48	0.013	**0.008**
Anthranilic acid (nM)	8.6 (6.7; 12.2)	7.9 (6.2; 10.6)	0.06	11.3 (8.6; 13.6)	9.9 (7.1; 14.6)	0.32	**0.002**	**0.005**
3-hydroxykynurenine (nM)	4.2 (3.4; 6.1)	4.6 (3.7; 6.8)	0.04	4.3 (3.3; 5.6)	4.7 (3.9; 7.3)	0.1	0.8	0.67
Picolinic acid (nM)	21.4 (16.9; 28.1)	26.4 (20.7; 34.8)	** < 0.001**	18.9 (14.7; 22.7)	22.2 (17.9; 28.3)	0.015	0.013	**0.004**
Quinolinic acid (nM)	29.1 (21.9; 40.2)	37.6 (27.9: 48.2)	** < 0.001**	34.0 (27.0; 47.8)	42.7 (29.8; 62.9)	0.11	0.011	0.14
KTR	20.2 (16.3; 23.2)	21.3 (17.3; 25.8)	0.1	19.9 (17.0; 24.5)	19.5 (16.8; 23.7)	0.62	0.65	0.16
KA/QA ratio	0.12 (0.08; 0.16)	0.10 (0.07; 0.13)	**0.001**	0.08 (0.06; 0.1)	0.08 (0.05; 0.1)	0.61	** < 0.001**	** < 0.001**

Data are presented as N (%) using χ^2^ test and median (Q1; Q3) using Mann–Whitney U test, significant differences *P* < .01 in bold*,* % = valid percent without missing, ^a^ = missing *APOE* data in 33 patients and 7 CU; missing BMI in 53 patients and all controls; missing IP-10 in 124 patients, † = comparison not possible due to inter-laboratory variability. Abbreviations: *AD* Alzheimer’s disease, *APOE* apolipoprotein E, *CSF* cerebrospinal fluid, *CU* cognitively unimpaired, *IP-10* interferon-γ induced protein 10, *KA* kynurenic acid, *KTR* kynurenine/tryptophan ratio, *NA* not available, *QA* quinolinic acid

### Sex differences in KP metabolite concentrations in CSF

In the CU cohort, there were no sex differences in KP metabolite levels in cross-sectional analyses. However, in the AD group men had significantly higher levels of KTR and several kynurenines including tryptophan, kynurenine, and QA, see Table 1. The KA/QA ratio was significantly higher in AD women (0.12) compared to AD men (0.10), *P* = 0.001.

AD women had significantly lower levels of AA (median = 8.6 nM) compared to CU women (median = 11.3 nM, *P* = 0.002). AD women had significantly higher KA/QA ratio (0.12) compared to CU women (0.08, *P* < 0.001). There were no further differences in kynurenine metabolite levels between AD women and CU women. AD men had significantly higher levels of KA (median = 3.7 nM) compared to CU men (median = 2.9 nM, *P* = 0.008). AD men and CU men differed significantly on levels of AA, Pic and the KA/QA ratio, see Table 1.

#### Interactions between sex and AD biomarkers on KP metabolite levels

In cross-sectional adjusted analyses including sex, Aβ_42_ and sex × Aβ_42_ as independent variables with age, *APOE* ε4 genotype, diagnosis and p-tau_181_ as covariates, neither sex nor the sex × Aβ_42_ interaction were associated with tryptophan, KP metabolites or ratios in the whole cohort, see Table 2. In the AD patients, male sex was associated with higher levels of Pic (β = 0.26, *P* < 0.01) and QA (β = 0.22, *P* < 0.01).

**Table 2:** The effects of sex × Aβ_42_ on tryptophan, KP metabolites and the KA/QA ratio

	**Trp**	**Kyn**	**KA**	**AA**	**3-HK**	**Pic**	**QA**	**KA/QA**
**A. Whole cohort**								
Diagnoses	-0.12	-0.02	0.18*	-0.25*	-0.01	0.2*	-0.11	0.23*
Age	0.10	0.30*	0.26*	0.20*	0.17*	0.01	0.45*	0.13
Sex	0.20	0.08	0.01	-0.16	0.08	0.19	0.21	-0.04
*APOE* ɛ4 genotype	0.01	-0.08	-0.01	0.03	0.04	-0.01	0.01	-0.00
Amyloid-β_42_	-0.02	-0.05	-0.09	0.04	-0.00	-0.09	-0.01	-0.08
P-tau_181_	-0.00	-0.03	0.22*	-0.04	0.00	0.08	0.02	0.23*
Sex x Amyloid-β_42_	-0.02	0.09	0.02	0.08	0.01	0.06	-0.04	0.02
Adjusted R^2^	0.06	0.12	0.13	0.08	0.02	0.07	0.27	0.1
**B. AD**								
Age	0.04	0.22*	0.22*	0.16*	0.19*	-0.01	0.42*	0.10
Sex	0.16	0.16	0.05	-0.12	0.07	0.26*	0.22*	-0.02
Amyloid-β_42_	0.07	0.05	0.04	-0.16	-0.00	-0.03	-0.01	0.04
P-tau_181_	0.07	-0.02	0.29*	-0.11	-0.00	0.04	0.04	0.30*
Sex x Amyloid-β_42_	0.03	-0.04	-0.01	0.06	0.03	0.01	0.07	-0.02
Adjusted R^2^	0.02	0.07	0.12	0.04	0.03	0.05	0.23	0.09
**C. CU**								
Age	0.36*	0.46*	0.35*	0.19	0.12	0.06	0.49*	0.21
Sex	0.20	0.11	0.04	-0.09	0.15	0.12	0.14	0.02
Amyloid-β_42_	0.00	-0.05	0.41*	0.19	0.13	0.17	-0.01	0.48*
P-tau_181_	-0.24	-0.17	0.10	-0.09	-0.01	-0.01	-0.00	0.11
Sex x Amyloid-β_42_	0.07	0.03	-0.30	-0.26	-0.14	-0.10	0.01	-0.37
Adjusted R^2^	0.15	0.18	0.17	0.02	-0.00	-0.02	0.23	0.11

^*^P < .01. significant associations in bold. Standardized β-coefficients are presented. Diagnoses: CU controls = 0, AD = 1; *APOE* ɛ4 genotype: neg = 0, pos = 1; Whole cohort (A): Amyloid-β_42_ and P-tau_181_ dichotomized according to laboratory-specific cut-off values: normal = 1, pathological = 2; AD patients (B) and CU controls (C): Amyloid-β_42_ and P-tau_181_ as continuous variables. Abbreviations: *3-HK* 3-hydroxykynurenine, *AA* anthranilic acid, *AD* Alzheimer’s disease, *APOE* apolipoprotein E, *CU* cognitively unimpaired, *KA* kynurenic acid, *Kyn* kynurenine, *Pic* picolinic acid, *Trp* trypthophan, *QA* quinolinic acid

In cross-sectional adjusted analyses including sex, p-tau_181_ and sex × p-tau_181_ as independent variables with age, *APOE* ε4 genotype, diagnosis and Aβ_42_ as covariates, male sex was a significant predictor of higher QA levels in the whole cohort (β = 0.22, *P* < 0.01), see Table 3. For the AD patients, age and Aβ_42_ were included as covariates and male sex was associated with higher levels of QA(β = 0.22, *P* < 0.01) and Pic (β = 0.26, *P* < 0.01), see Table 3. The sex × p-tau_181_ interaction element was not associated with any KP metabolites, tryptophan or the KA/QA ratio.

**Table 3:** The effects of sex × p-tau_181_ on tryptophan, KP metabolites and the KA/QA ratio

	**Trp**	**Kyn**	**KA**	**AA**	**3-HK**	**Pic**	**QA**	**KA/QA**
**A. Whole cohort**								
Diagnoses	-0.12	-0.02	0.18*	-0.25*	-0.01	0.20*	-0.11	0.23*
Age	0.11	0.30*	0.26*	0.19*	0.17*	0.00	0.45*	0.13
Sex	0.18	0.14	0.07	-0.03	0.19	0.19	0.22*	0.02
*APOE* ɛ4 genotype	0.01	-0.08	-0.01	0.03	0.03	0.00	0.01	-0.01
Amyloid-β_42_	-0.03	-0.01	-0.07	0.09	0.02	-0.07	-0.02	-0.06
P-tau_181_	-0.01	-0.02	0.26*	0.02	0.09	0.04	0.05	0.27*
Sex x P-tau_181_	0.01	-0.01	-0.08	-0.11	-0.16	0.08	-0.06	-0.08
Adjusted R^2^	0.06	0.12	0.13	0.08	0.03	0.07	0.27	0.11
**B. AD**								
Age	0.04	0.22*	0.22*	0.16*	0.18*	-0.01	0.41*	0.10
Sex	0.16*	0.16*	0.04	-0.12	0.07	0.26*	0.22*	-0.02
Amyloid-β_42_	0.08	0.01	0.02	-0.13	-0.01	-0.01	0.03	0.01
P-tau_181_	0.10	0.02	0.34*	-0.09	0.08	0.03	0.07	0.34*
Sex x P-tau_181_	-0.07	-0.06	-0.09	-0.04	-0.16	0.03	-0.08	-0.06
Adjusted R^2^	0.03	0.07	0.12	0.04	0.04	0.05	0.23	0.09
**C. CU**								
Age	0.36*	0.46*	0.33*	0.18	0.11	0.05	0.49*	0.19
Sex	0.20	0.11	0.05	-0.09	0.15	0.12	0.14	0.02
Amyloid-β_42_	0.05	-0.03	0.21	0.02	0.03	0.09	0.00	0.22
P-tau_181_	-0.34	-0.21	0.27	0.12	-0.09	-0.08	-0.07	0.32
Sex x P-tau_181_	0.12	0.05	-0.23	-0.28	0.09	0.08	0.08	-0.28
Adjusted R^2^	0.15	0.18	0.15	0.02	-0.01	-0.02	0.23	0.08

^*^P < .01. significant associations in bold. Standardized β- coefficients are presented. Diagnoses: CU controls = 0, AD = 1; *APOE* ɛ4 genotype: neg = 0, pos = 1; Whole cohort (A): Amyloid-β_42_ and P-tau_181_ dichotomized according to laboratory-specific cut-off values: normal = 1, pathological = 2; AD patients (B) and CU controls (C): Amyloid-β_42_ and P-tau_181_ as continuous variables. Abbreviations: *3-HK* 3-hydroxykynurenine, *AA* anthranilic acid, *AD* Alzheimer’s disease, *CU* cognitively unimpaired, *KA* kynurenic acid, *Kyn* kynurenine, *Pic* picolinic acid, *Trp* trypthophan, *QA* quinolinic acid

Rerunning these models for the subset of AD patients with BMI as a covariate did not affect these results (n = 258), although BMI was associated with significantly higher levels of kynurenine (β = 0.16), KA (β = 0.30), QA (β = 0.19) and higher KA/QA ratios (β = 0.26), all *P* < 0.001.

#### Interactions between sex and inflammation biomarkers on KP metabolite levels

**Fig. 2:**
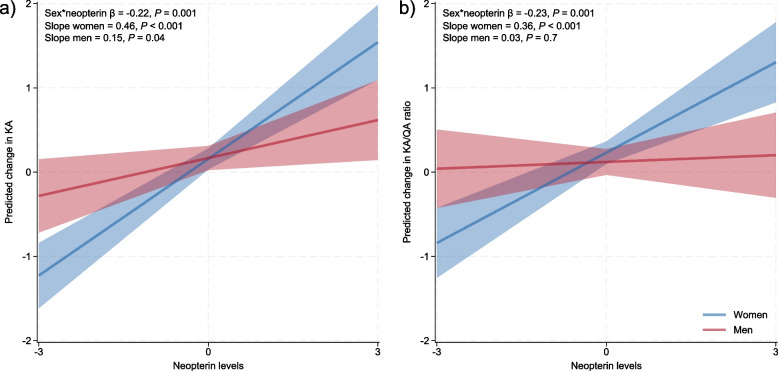
Interaction between neopterin and sex on KA levels and the KA/QA ratio in AD. Marginsplot with 95% confidence intervals of predicted change in **a**) KA and **b**) KA/QA ratio for men and women across levels of z-scored neopterin ranging from three standard deviations below the mean to three standard deviations above the mean. The interaction is adjusted for main effects of sex and neopterin as well as age, CSF Aβ_42_ and CSF p-tau_181_ levels. Abbreviations: Aβ: amyloid-β; AD: Alzheimer’s disease; CSF: cerebrospinal fluid; KA: kynurenic acid; p-tau: phosphorylated tau; QA: quinolinic acid

In cross-sectional adjusted analyses including sex, neopterin and sex × neopterin as independent variables with age, *APOE* ε4 genotype, Aβ_42_, p-tau_181_ and diagnosis as covariates, male sex and high neopterin were significantly associated with increased levels of several kynurenines in the whole cohort, see Table 4; this association was driven by the AD patients, as there were no significant associations with sex or neopterin in the CU group. Moreover, there was a significant interaction of neopterin and sex on the KA/QA ratio (β = -0.19, *P* < 0.01) and a trend for KA (β =—0.16, *P* = 0.01). These interactions were significant in the AD group for both KA (β = -0.22, *P* < 0.01) and the KA/QA ratio (β = -0.23, *P* < 0.01), but not in the CU group, see Table 4. As such, increased levels of neopterin were more strongly associated with increased levels of KA and higher KA/QA ratios in women than in men, see Fig. 2. Indeed, for low levels of neopterin AD women are predicted to have lower levels of KA and lower KA/QA ratios than AD men, whereas the situation is inverse for high levels, see Fig. 2. Including BMI as a covariate in the AD patient models (for a subset of patients, n = 258) did not affect the interactions between sex and neopterin.

**Table 4:** The effects of sex × neopterin on tryptophan, KP metabolites and the KA/QA ratio

	**Trp**	**Kyn**	**KA**	**AA**	**3-HK**	**Pic**	**QA**	**KA/QA**
**A. Whole cohort**								
Diagnoses	-0.09	0.12	0.28*	-0.23*	0.05	0.22*	0.01	0.30*
Age	0.06	0.15*	0.17*	0.17*	0.11	-0.02	0.32*	0.08
Sex	0.18*	0.15*	0.03	-0.10	0.09	0.24*	0.19*	-0.02
*APOE* ɛ4 genotype	0.01	-0.09	-0.03	0.04	0.04	-0.01	0.00	-0.02
Amyloid-β_42_	-0.02	0.02	-0.06	0.08	0.01	-0.06	0.00	-0.06
P-tau_181_	-0.02	-0.10	0.17*	-0.05	-0.03	0.07	-0.05	0.20*
Neopterin (log10)	0.07	0.47*	0.39*	0.08	0.20*	0.05	0.40*	0.30*
Neopterin x sex	0.09	0.00	-0.16	-0.05	-0.01	0.05	0.01	-0.19*
Adjusted R^2^	0.07	0.29	0.20	0.08	0.05	0.07	0.39	0.14
**B. AD**								
Age	0.02	0.08	0.12	0.12	0.14	-0.03	0.30*	0.03
Sex	0.16*	0.14*	0.01	-0.13	0.05	0.27*	0.20*	-0.06
Amyloid-β_42_	0.09	-0.03	0.00	-0.13	0.00	-0.03	0.00	-0.01
P-tau_181_	0.04	-0.13	0.20*	-0.15	-0.05	0.03	-0.07	0.24*
Neopterin (log10)	0.09	0.50*	0.48*	0.16	0.23*	0.02	0.44*	0.37*
Neopterin x sex	0.00	-0.05	-0.22*	-0.04	-0.05	0.07	-0.07	-0.23*
Adjusted R^2^	0.03	0.26	0.24	0.06	0.05	0.06	0.36	0.15
**C. CU**								
Age	0.22	0.25*	0.31*	0.21	-0.08	0.00	0.29*	0.22
Sex	0.11	-0.16	0.12	-0.15	0.15	0.14	0.14	0.11
Amyloid-β_42_	0.04	-0.04	0.20	0.00	0.02	0.09	-0.01	0.21
P-tau_181_	-0.24*	-0.17	0.08	-0.11	-0.02	-0.01	0.00	0.09
Neopterin (log10)	0.00	0.34	0.16	-0.15	0.21	0.09	0.24	0.13
Neopterin x sex	0.31	0.10	-0.13	0.09	0.20	0.02	0.22	-0.23
Adjusted R^2^	0.21	0.31	0.13	-0.01	0.10	-0.02	0.37	0.06

^*^P < .01. significant associations in bold. Standardized β- coefficients are presented. Diagnoses: CU controls = 0, AD = 1; *APOE* ɛ4 genotype: neg = 0, pos = 1; Whole cohort (A): Amyloid-β_42_ and P-tau_181_ dichotomized according to laboratory-specific cut-off values: normal = 1, pathological = 2; AD patients (B) and CU controls (C): Amyloid-β_42_ and P-tau_181_ as continuous variables. Abbreviations: *3-HK* 3-hydroxykynurenine, *AA* anthranilic acid, *AD* Alzheimer’s disease, *CU* cognitively unimpaired, *KA* kynurenic acid, *Kyn* kynurenine, *Pic* picolinic acid, *Trp* trypthophan, *QA* quinolinic acid

In cross-sectional analyses including sex, IP-10 and sex × IP-10 as independent variables, there were trends in the same direction for the whole cohort on KA levels (β = -0.17, *P* = 0.03) and the KA/QA ratio (β = -0.18, *P* = 0.02), and a significant interaction on KA levels (β = -0.23, *P* = 0.008) and a trend on the KA/QA ratio (β = -0.19, *P* = 0.04) for the AD patients, see Supplementary Table S1.

### Interactions between sex and kynurenine metabolites on clinical progression of AD

In the longitudinal analysis, there were 238 patients with at least one follow-up consultation; average follow-up time was 2.01 years and the number of follow-up consultations ranged from 1 to 7 with an average of 2.4. For women (n = 136) the average follow-up time was 2.06 years (average number of follow-up visits = 2.4, range 1–7) and for men (n = 102) the average follow-up time was 1.96 years (average number of follow-up visits = 2.2, range 1–7). There were no significant interactions between sex and baseline KA or KA/QA ratio on clinical progression measured by the CDR-SB in the AD patients, see Table 5. No other metabolites were significantly associated with clinical progression, but there were significant interactions between sex and 3-HK (β = 1.10) and QA (β = 0.82) on baseline CDR-scores, see Supplementary Table S2. The interactions with time were not affected by adjusting for CSF Aβ_42_, p-tau_181_ and neopterin. Moreover, neopterin itself was not a significant predictor of clinical progression in analyses adjusted for age, CSF Aβ_42_ and p-tau_181_ (data not shown).

**Table 5:** Interaction effects between sex and baseline CSF KA and KA/QA ratio on clinical progression in AD

	**KA**		**KA/QA**	
	Coefficient (95% CI)	*P*	Coefficient (95% CI)	*P*
**Age**	0.02 (-0.03; 0.06)	0.47	0.02 (-0.03; 0.06)	0.48
**Metabolite**	-0.20 (-0.51; 0.12)	0.22	0.30 (-0.04; 0.64)	0.08
**Time**	1.76 (1.50; 2.02)	< 0.01	1.76 (1.50; 2.02)	< 0.01
**Metabolite × time**	-0.18 (-0.41; 0.05)	0.12	-0.12 (-0.36; 0.11)	0.31
**Sex**	0.03 (-0.56; 0.61)	0.93	0.10 (-0.49; 0.69)	0.74
**Sex × metabolite**	0.34 (-0.33; 1.02)	0.32	-0.27 (-0.87; 0.33)	0.38
**Sex × time**	-0.34 (-0.75; 0.06)	0.10	-0.32 (-0.73; 0.09)	0.13
**Sex × metabolite × time**	0.17 (-0.31; 0.65)	0.48	0.19 (-0.25; 0.64)	0.40

Linear mixed models assessing the interaction between sex and baseline CSF KA or the KA/QA ratio on clinical progression (change in CDR-SB over time) in the AD patients. Random effects included time from baseline and intercept. Abbreviations: *CDR-SB* clinical dementia rating sum of boxes, *KA* kynurenic acid, *QA* Quinolinic acid

### Sensitivity analyses

All analyses were rerun excluding the patients on anti-dementia medications at baseline (n = 28). After excluding these patients, the levels of tryptophane did no longer differ significantly between AD men and AD women, and the levels of AA did not differ between AD men and CU men. In the regression analyses, the neopterin × sex interaction was no longer significant in analyses of the whole cohort, however it remained significant in the AD cohort (β =-0.23, *P* < 0.01). No other biomarker × sex interactions were affected.

## Discussion

We found sex differences in several KP metabolites in the AD group, while there were no sex differences in the CU group. Furthermore, we found in sex-stratified analyses that the neuroprotective metabolites KA and Pic were exclusively increased in AD men, whilst AA and the KA/QA ratio were altered in AD in both men and women. To investigate these sex differences, we explored interactions between CSF Aβ_42,_ p-tau_181_, neopterin and sex. We found sex-specific associations with neopterin on both KA levels and the KA/QA ratio in the AD group, with increasing neopterin being associated with a steeper increase in KA and the KA/QA ratio in AD women. There were no sex-specific associations between any KP metabolites and clinical progression.

Our finding of sex differences in KP metabolites in CSF only in the AD patients contrasts with previous work showing sex differences in plasma metabolites in cognitively healthy individuals [8, 43, 44]. However, this could be due to differences in cohort age or between CSF and plasma measurements, factors known to influence KP metabolite levels [17, 45]. Within the AD group, there were sex differences tryptophan and all measured KP metabolites except KA and AA, but there were no sex differences in KTR. The fact that there are no sex differences in KTR, whilst both tryptophan and kynurenine are increased in men, suggests a general increase in KP activity in men, possibly as a consequence of increased tryptophan availability [33]. It has previously been suggested that higher concentrations of tryptophan and KP metabolites in men are related to higher protein intake and/or turnover, explained by higher muscle mass in men [33]. However, here we observed increased KP metabolites exclusively in men compared to women only in the AD group, suggesting that dysregulation of KP metabolism in AD patients might be influenced by sex. Specifically, increased levels of all KP metabolites except KA and AA point to possible sex differences in kynurenine 3-monooxygenese (KMO) activity, which catalyses the conversion of kynurenine to 3-hydroxykynurenine. Indeed, an upregulation of KMO shifts the KP pathway to the QA branch and is common under inflammatory conditions [46]. To determine if sex differences in KP metabolism are connected to AD pathophysiology, sex-specific associations between KMO expression and AD biomarkers should be examined.

In our cohort more metabolites differed significantly between CU and AD in men than in women, suggesting a more consistent change in KP metabolites in men, in contrast to previous research reporting female-specific increases in both neuroprotective KA and neurotoxic QA in AD patients compared to controls [20]. However, while we found male-specific increases in the neuroprotective metabolites Pic and KA, it should be noted that we observed a similar trend in women. Increased levels of CSF KA in AD have been shown previously and high levels of Pic have been found to inversely correlate with CSF levels of p-tau [17, 47]. The downstream effects of increased KA and Pic in AD men are uncertain. KA is primarily produced in the astrocytes in response to neuroinflammation and is believed to be compensatory mechanism that reduces neurotoxicity [12]. Similarly, Pic is known to inhibit the neurotoxic effects of QA [48]. While generally considered neuroprotective, high levels of KA and Pic may also have negative effects on cognition [5, 12, 49], possibly modified by *APOE* ε4 genotype [50]. Further research should explore sex differences in these markers in a larger AD cohort to elucidate whether the increase in KA and Pic in AD is unique to men.

To explore sex-specific associations between the KP and inflammation, we examined interactions between sex, AD biomarkers and neopterin on tryptophan, KP metabolites and the KA/QA ratio. We found no sex differences in the AD biomarkers in the AD or CU group, and we found no sex-specific interactions between Aβ_42_, p-tau_181_ and any of the outcome variables. However, we found that sex and neopterin interacted on the levels of KA and the KA/QA ratio in the AD group. This suggests sex differences in the interaction between inflammation and KP metabolism in these patients, although previous work proposes that inflammation is not the key mechanism linking KP to AD pathology [17]. We found that low levels of neopterin were associated with lower levels of KA for women than for men, with a relatively larger increase in KA for women with increasing levels of neopterin. Furthermore, we found a significant interaction between sex × IP-10 on KA levels in a subset of the AD cohort. Importantly, both neopterin production, IP-10 levels and tryptophan catabolism via the KP are stimulated by the pro-inflammatory cytokine IFN-γ; during immune activation IFN-γ stimulates the production of neopterin by macrophages and increases the conversion of tryptophan to kynurenine by up-regulating the enzyme indoleamine 2,3-dioxygenase [32, 33]. Our results suggest that the KP might be upregulated in response to lower levels of inflammation in men than women, with higher levels of inflammation yielding a stronger increase in KA levels and the KA/QA ratio in women. One possible interpretation of this finding is that men produce relatively more KMO in response to increased inflammation, shifting the KP towards the QA branch and resulting in less KA, *i.e.* shifting KP metabolism from the neuroprotective (KA) branch to the neurotoxic (QA) branch.

Interestingly, we also found sex differences in neopterin levels in the CU group with higher levels in women but not in the AD group. This has previously been found in middle aged individuals but not in older persons; sex differences in chronic immune activation have been put forth as a potential explanation for this finding [8, 51]. Increased levels of CSF IFN-γ have also been found in women regardless of AD pathology [52]. In contrast to previous research in plasma, we found higher levels of CSF neopterin in healthy controls compared to AD patients [53]; the association between plasma and CSF levels of neopterin appear to vary across diseases and needs to be explored in AD. Neopterin levels were not increased in AD compared to controls, despite the considerable role of neuroinflammation in AD pathophysiology; this is in line with previous research showing unaltered neopterin levels in AD and other dementias [54]. Furthermore, lower CSF IFN-γ have been reported for CU individuals with amyloid pathology compared to CU individuals with normal biomarkers [52]. However, we did find increased levels of IP-10 in AD patients; IP-10 is another indicator of IFN-γ activity which has previously been linked to dysregulation of the KP [55]. This suggests a complex relationship between IFN-γ activity and neuroinflammation in AD; indeed, AD pathology is associated with several markers of microglia and astrocyte hyper- and hypoactivity which may not be completely reflected by IFN-γ related markers such as CSF neopterin and IP-10 levels [52]. With regards to sex differences in immune activation and the KP pathway, it would be of interest to explore interactions with biomarkers of astrogliosis such as plasma levels of glial fibrillary acid protein (GFAP) [56], especially given the role of astrocytes in KA synthesis.

There were no significant associations between sex and baseline KP metabolite levels or the KA/QA ratio on clinical progression measured by the CDR-SB in the AD patients. We have previously shown in this cohort that higher levels of KA are significantly associated with slower clinical progression [19]. Surprisingly, while we found sex-specific interactions between KA and neopterin, this did not correspond to sex differences in the association between KA and clinical progression. Moreover, neopterin was not itself associated with clinical progression. This suggests that while there appears to be sex differences in KA metabolism in response to inflammation in AD, the effects of KA on progression are not affected. It would be of interest to explore whether sex-specific associations with KA and KA/QA extend to other markers of neuroinflammation known to influence AD progression, such as CSF levels of soluble triggering receptor expressed on myeloid cells 2 (sTREM2) [57, 58]. Moreover, we did find significant interactions between sex × 3-HK and sex × QA on baseline CDR, suggesting sex differences in the association between these neurotoxic metabolites and cognitive and functional status. The association between the metabolites on disease progression at earlier (preclinical) disease stages should be explored in relation to sex.

Beyond its role in neuroinflammation, the KP is the only de novo NAD^+^ synthetic pathway. NAD^+^ plays a critical role in mitochondrial energy production and stimulates mitophagy which specifically recognises and degrades damaged mitochondria, leading to neuronal protection [59]. Compromised mitochondrial homeostasis, due to defective mitophagy, during ageing likely contributes to sex-specific metabolic disturbance, inflammation, and higher AD risk in women. Mitochondrial metabolic dysfunction is a common feature of neurodegenerative disease, and there is an intertwined interaction among metabolic imbalance, mitochondrial damage and neuronal dysfunction. Of note, higher autophagy/mitophagy increases cellular NAD^+^ and resilience [60]. Interestingly, fine-tuning the NAD^+^ metabolic pathway as well as restoration of mitophagy inhibits pathology and memory loss in different AD animal models [59, 61]. In line with an important role of defective NAD^+^-mitophagy axis in AD, there are sex differences in mitochondrial function and mitophagy [62, 63]. Indeed, studies in AD mice and AD postmortem human samples have illustrated sex-specific metabolic alterations in AD, correlating with amyloid and tau pathologies [63]. Thus, a sex-specific vulnerability against NAD^+^-depletion-induced mitochondrial damage may contribute to higher AD risk in women.

A strength of our study is the large well-characterised cohorts of both clinical AD patients and CU controls. All included patients and controls were assessed with the same cognitive tests, most were followed up for several years and all had available AD biomarker data from baseline. However, it is a limitation of our study that the AD biomarkers Aβ_42_ and p-tau_181_ could not be directly compared between the AD and CU groups as these were analysed in different laboratories. Furthermore, while it has recently been shown that CSF AD biomarkers are influenced by kidney function [64], we were unable to control for variations in kidney function at the time of lumbar puncture. Both body composition and race are known to influence the levels of KP metabolites and neopterin [51, 65]; while we found that BMI, a proxy for body composition, did not affect our conclusions in the AD group, we could not account for potential differences in body composition between the AD and CU groups. Moreover, as our cohort is predominantly Caucasian, these results should be replicated in a more diverse cohort. Further research should explore the influence of sex hormones on KP metabolism in AD, as sex hormones have been shown to influence both KP metabolism and neopterin production through attenuated IFN-γ activity [44]; moreover, it should be examined whether IFN-γ mediates the interaction between neopterin, KA and sex. Of relevance to our cohort of postmenopausal women, it is unclear whether KP activity is altered after menopause because of the plummet in oestrogen, while relatively stable testosterone levels in similarly aged men may confer neuroprotection [29]. Additionally, the links between KP pathway dysfunction, damaged mitochondria and defective mitophagy should be studied in future animal and human experiments.

## Conclusions

In our cohort sex differences in KP metabolites are present in AD patients but not in healthy controls. We found that more KP metabolites were significantly altered in AD men compared to CU men, and a trend in the same direction was evident in AD women. These sex differences could potentially be driven by sex-specific changes in KP metabolism in response to increased neuroinflammation, as we found sex-specific associations between KA and the KA/QA ratio with neopterin, a marker of general inflammation. However, these results do not appear to influence the protective effect of KA on clinical progression. Further research should explore the roles of sex hormones and mitophagy in the complex interplay between AD pathophysiology, KP metabolism and neuroinflammation.

### Supplementary Information

Supplementary Material 1.

## Data Availability

The data that support the findings of this study are not openly available due to reasons of sensitivity and legal restrictions imposed by the registry owners and the ethical committee. The clinical data on the memory clinic patients may be requested from the Norwegian Registry of Persons Assessed for Cognitive Symptoms (NorCog) at e-mail: post@aldringoghelse.no. The demographic data for the CU controls and results of the KP analyses are available upon reasonable request to the authors. All data accessibility is dependent on the approval from the REC South East, contact at e-mail: post@helseforskning.etikkom.no.

## Abbreviations

AA: Anthranilic acid
Aβ: Amyloid-β
AD: Alzheimer's disease
*APOE*: Apolipoprotein E
CDR-SB: Clinical dementia rating scale—sum of boxes
CSF: Cerebrospinal fluid
CU: Cognitively unimpaired
ELISA: Enzyme-linked immunosorbent assay
IFN-γ: Interferon-γ
IP-10: Interferon-γ induced protein 10
3-HK: 3-Hydroxykynurenine
3-HAA: 3-Hydroxyanthranilic acid
KA: Kynurenic acid
KMO: Kynurenine 3-monooxygenese
KP: Kynurenine pathway
KTR: Kynurenine/tryptophane-ratio
MCI: Mild cognitive impairment
NMDA: *N*-Methyl-D-aspartate
p-tau: Phosphorylated tau
QA: Quinolinic acid
sTREM2: Soluble triggering receptor expressed on myeloid cells 2
TRP: Tryptophan
XA: Xanthurenic acid
