# Supplementary material for: Sex-specific associations of kynurenic acid with neopterin in Alzheimer’s disease
Source: Alzheimers Res Ther. 2024 Jul 27;16:167. doi: 10.1186/s13195-024-01531-7 (PMC11282793; doi:10.1186/s13195-024-01531-7)
Supplement: Supplementary file 1 — Supplementary Material 1. [file 13195_2024_1531_MOESM1_ESM.docx]

Sex-specific associations of kynurenic acid with neopterin in Alzheimer’s disease: Supplementary material

Anne-Brita Knapskog^1^, Trine Holt Edwin^1^, Per Magne Ueland^2^, Arve Ulvik^2^, Evandro Fei Fang^3, 4^, Rannveig Sakshaug Eldholm^5, 6^, Nathalie Bodd Halaas^7, 12^, Lasse M. Giil^8, 9^, Ingvild Saltvedt^5, 6^, Leiv Otto Watne^7, 10, 11^, Mari Aksnes^12*^

**Author affiliations:**

^1^ Department of Geriatric Medicine, Oslo University Hospital, 0450 Oslo, Norway

^2^ Bevital AS, 5021 Bergen, Norway

^3^ Department of Clinical Molecular Biology, University of Oslo and Akershus University Hospital, 1478 Lørenskog, Norway

^4^ The Norwegian Centre on Healthy Ageing (NO-Age), Oslo, Norway

^5^ Department of Neuromedicine and Movement Science, Norwegian University of Science and Technology, 7491Trondheim, Norway

^6^ Department of Geriatric Medicine, St. Olavs Hospital, Trondheim University Hospital, 7006 Trondheim, Norway

^7^ Oslo Delirium Research Group, Oslo University Hospital, 0450 Oslo, Norway

^8^ Neuro-SysMed, Department of Internal Medicine, Haraldsplass Deaconess Hospital, 5892 Bergen, Norway

^9^ Department of Clinical Science, University of Bergen, 5021 Bergen, Norway

^10^ Institute of Clinical Medicine, Campus Ahus, University of Oslo, 1478 Lørenskog, Norway

^11^ Department of Geriatric Medicine, Akershus University Hospital, 1478 Lørenskog, Norway

^12^ Department of Geriatric Medicine, University of Oslo, 0315 Oslo, Norway

* Correspondence to: Mari Aksnes, e-mail: mari.aksnes@medisin.uio.no, telephone number: +47 47303885, postal address: OUS HF Ullevål Hospital, PO box 4956 Nydalen, 0424 Oslo

# Supplementary methods

**Biochemical analysis of interferon-γ induced protein 10 (IP-10)**

For all CU controls (n = 105) and a subset of the AD patients (n = 187), CSF levels of interferon-γ induced protein 10 (IP-10) were analysed together with eight other cytokines using a custom-made nine-plex kit (Cat No. 12014058, Bio-Rad Laboratories, Hercules, CA) containing eotaxin-1, granulocyte colony-stimulating factor (G-CSF), interleukin (IL)-6, IL-7, IL-8, IP-10, monocyte chemoattractant protein 1 (MCP-1), macrophage inflammatory protein (MIP)-1α, and MIP-1β. Briefly, a solution of 10% bovine serum albumin (Cat No. A5403-50G, lot SLBL9495V, Sigma Aldrich, St. Louis, MO) in PBS (pH 7.4, Gibco Cat No. 10010–015, lot 2062123, Thermo Fisher Scientific, Waltham, MA) was added to the CSF samples to a concentration of 0.5% before vortexing. Samples were centrifuged at 10 000×g for 10 min at 4 °C and 50 μL of the supernatant was loaded onto the assay plate. Cytokine levels were measured on a Luminex IS 200 instrument (Bio-Rad).

# Supplementary tables

**Supplementary Table S1. The effects of sex × IP-10 on tryptophan, KP metabolites and the KA/QA ratio**

| **A. Whole cohort** | | | | |  | | | | |
| --- | --- | --- | --- | --- | --- | --- | --- | --- | --- |
|  | **Trp** | **Kyn** | **KA** | **AA** | | **3-HK** | **Pic** | **QA** | **KA/QA** |
| Diagnoses | -0.05 | -0.13 | 0.16* | **-0.45*** | | 0.01 | **0.31*** | **-0.21*** | **0.24*** |
| Age | 0.09 | **0.30*** | **0.29*** | 0.12 | | 0.15 | 0.03 | **0.42*** | 0.17 |
| Sex | 0.16 | 0.14 | 0.01 | -0.8 | | 0.06 | **0.21*** | **0.18*** | -0.04 |
| *APOE* ɛ4 genotype | 0.00 | -0.07 | -0.06 | 0.06 | | -0.02 | -0.04 | -0.01 | -0.04 |
| Amyloid-β_42_ | -0.06 | 0.02 | -0.06 | 0.07 | | -0.04 | -0.02 | -0.03 | -0.06 |
| P-tau_181_ | -0.02 | -0.06 | **0.16*** | 0.09 | | 0.01 | -0.01 | -0.01 | 0.17 |
| IP-10 | 0.00 | **0.37*** | **0.34*** | 0.14 | | 0.11 | -0.12 | **0.32*** | **0.26*** |
| IP-10 x sex | 0.07 | -0.04 | -0.17 | -0.07 | | -0.04 | 0.16 | -0.02 | -0.18 |
| Adjusted R^2^ | 0.03 | 0.24 | 0.22 | 0.13 | | 0.01 | 0.10 | 0.37 | 0.16 |
| **B. AD** |  |  |  |  | |  |  |  |  |
|  | **Trp** | **Kyn** | **KA** | **AA** | | **3-HK** | **Pic** | **QA** | **KA/QA** |
| Age | -0.04 | **0.20*** | **0.28*** | 0.12 | | **0.19*** | 0.01 | **0.38*** | 0.18 |
| Sex | **0.12*** | **0.21*** | 0.08 | -0.08 | | -0.00 | **0.25*** | **0.26*** | 0.00 |
| Amyloid-β_42_ | 0.09 | -0.05 | -0.02 | -0.10 | | 0.05 | -0.07 | 0.02 | -0.03 |
| P-tau_181_ | 0.07 | -0.07 | **0.21*** | -0.06 | | 0.00 | -0.03 | -0.02 | **0.24*** |
| IP-10 | 0.06 | **0.47*** | **0.36*** | 0.19 | | 0.06 | -0.10 | **0.41*** | 0.22 |
| IP-10 x sex | -0.15 | -0.17 | **-0.23*** | -0.07 | | -0.01 | 0.04 | -0.10 | -0.19 |
| Adjusted R^2^ | 0.00 | 0.22 | 0.23 | 0.03 | | 0.01 | 0.05 | 0.36 | 0.12 |
| **C. CU** |  |  |  |  | |  |  |  |  |
|  | **Trp** | **Kyn** | **KA** | **AA** | | **3-HK** | **Pic** | **QA** | **KA/QA** |
| Age | **0.35*** | **0.40*** | **0.30*** | 0.19 | | 0.08 | -0.01 | **0.44** | 0.18 |
| Sex | **0.29*** | 0.16 | 0.02 | -0.13 | | 0.19 | 0.24 | 0.17 | -0.04 |
| Amyloid-β_42_ | 0.04 | -0.17 | 0.20 | 0.00 | | 0.03 | 0.09 | 0.00 | 0.20 |
| P-tau_181_ | -0.24 | -0.03 | 0.08 | -0.11 | | -0.02 | -0.02 | 0.00 | 0.09 |
| IP-10 | -0.27 | 0.24 | 0.28 | 0.09 | | 0.07 | -0.08 | 0.25 | 0.26 |
| IP-10 x sex | 0.29 | 0.08 | -0.13 | -0.12 | | 0.08 | 0.31 | 0.00 | -0.20 |
| Adjusted R^2^ | 0.17 | 0.26 | 0.17 | -0.01 | | -0.00 | 0.02 | 0.28 | 0.07 |

*P < .01. significant associations in **bold**. Standardized β-coefficients are presented. Diagnoses: CU controls = 0, AD = 1; *APOE* ɛ4 genotype: neg = 0, pos = 1; Whole cohort (A): Amyloid-β_42_ and P-tau_181_ dichotomized according to laboratory-specific cut-off values: normal = 1, pathological = 2; AD patients (B) and CU controls (C): Amyloid-β_42_ and P-tau_181_ as continuous variables. **Abbreviations:** 3-HK: 3-hydroxykynurenine; AA: anthranilic acid; AD: Alzheimer’s disease; *APOE*: apolipoprotein E; CU: cognitively unimpaired; IP-10: interferon-γ induced protein 10. KA: kynurenic acid; Kyn: kynurenine; Pic: picolinic acid; Trp: trypthophan; QA: quinolinic acid

**Supplementary Table S2. Interaction effects between sex and baseline CSF KP metabolites on clinical progression in AD**

|  | **Tryptophan** | **Kynurenine** | **Anthranilic acid** | **3-HK** | **Picolinic acid** | **Quinolinic acid** |
| --- | --- | --- | --- | --- | --- | --- |
| **Age** | 0.01 (-0.03; 0.06) | 0.02 (-0.03; 0.06) | 0.01 (-0.04; 0.06) | 0.01 (-0.03; 0.06) | 0.01 (-0.03; 0.06) | 0.02 (-0.02; 0.07) |
| **Metabolite** | -0.32 (-0.72; 0.09) | -0.25 (-0.61; 0.12) | 0.17 (-0.16; 0.50) | -0.35 (-0.72; 0.03) | -0.11 (-0.57; 0.36) | -0.47 (-0.82; -0.12)* |
| **Time** | 1.74 (1.48; 2.00)* | 1.73 (1.47; 1.99)* | 1.74 (1.48; 2.01)* | 1.74 (1.49; 2.01)* | 1.75 (1.48; 2.02)* | 1.73 (1.47; 1.99)* |
| **Metabolite × time** | -0.03 (-0.32; 0.25) | -0.24 (-0.50; 0.02) | 0.01 (-0.23; 0.24) | -0.05 (-0.31; 0.22) | 0.00 (-0.31; 0.32) | -0.16 (-0.39; 0.08) |
| **Sex** | 0.06 (-0.52; 0.64) | 0.01 (-0.57; 0.60) | 0.10 (-0.47; 0.68) | -0.00 (-0.56; 0.56) | 0.16 (-0.44; 0.75) | -0.03 (-0.61; 0.54) |
| **Sex × metabolite** | 0.48 (-0.09; 1.05) | 0.44 (-0.11; 0.99) | 0.20 (-0.38; 0.68) | 1.10 (0.58; 1.63)* | -0.23 (-0.85; 0.38) | 0.82 (0.30; 1.34)* |
| **Sex × time** | -0.32 (-0.73; 0.09) | -0.31 (-0.71; 0.10) | -0.34 (-0.75; 0.07) | -0.30 (-0.70; 0.10) | -0.38 (-0.79; 0.04) | -0.29 (-0.69; 0.12) |
| **Sex × metabolite × time** | 0.04 (-0.36; 0.44) | 0.18 (-0.23; 0.59) | 0.05 (-0.52; 0.06) | 0.18 (-0.56; 0.20) | 0.19 (-0.24; 0.62) | 0.02 (-0.37; 0.41) |

Linear mixed models assessing the interaction between sex and baseline CSF kynurenine metabolites on clinical progression (change in CDR-SB over time) in the AD patients. Random effects included time from baseline and intercept. Significant effects (*P* < 0.01) are noted with an * and grey background. **Abbreviations:** 3-HK: 3-hydroxykynurenine; CDR-SB: clinical dementia rating sum of boxes; KA: kynurenic acid; KP: kynurenine pathway; QA, Quinolinic acid
